# Role of Polycomb Complexes in Normal and Malignant Plasma Cells

**DOI:** 10.3390/ijms21218047

**Published:** 2020-10-28

**Authors:** Emmanuel Varlet, Sara Ovejero, Anne-Marie Martinez, Giacomo Cavalli, Jerome Moreaux

**Affiliations:** 1 Institute of Human Genetics, UMR 9002 Centre National de la Recherche Scientifique, University of Montpellier, Montpellier, 34396 Montpellier, France; emmanuel.varlet@igh.cnrs.fr (E.V.); sara.ovejero-merino@igh.cnrs.fr (S.O.); anne-marie.martinez@igh.cnrs.fr (A.-M.M.); giacomo.cavalli@igh.cnrs.fr (G.C.); 2 Department of Biological Hematology, CHU Montpellier, 34295 Montpellier, France; 3 UFR Medicine, University of Montpellier, 34003 Montpellier, France; 4 Institut Universitaire de France (IUF), 75005 Paris, France

**Keywords:** epigenetics, plasma cell differentiation, multiple myeloma, polycomb

## Abstract

Plasma cells (PC) are the main effectors of adaptive immunity, responsible for producing antibodies to defend the body against pathogens. They are the result of a complex highly regulated cell differentiation process, taking place in several anatomical locations and involving unique genetic events. Pathologically, PC can undergo tumorigenesis and cause a group of diseases known as plasma cell dyscrasias, including multiple myeloma (MM). MM is a severe disease with poor prognosis that is characterized by the accumulation of malignant PC within the bone marrow, as well as high clinical and molecular heterogeneity. MM patients frequently develop resistance to treatment, leading to relapse. Polycomb group (PcG) proteins are epigenetic regulators involved in cell fate and carcinogenesis. The emerging roles of PcG in PC differentiation and myelomagenesis position them as potential therapeutic targets in MM. Here, we focus on the roles of PcG proteins in normal and malignant plasma cells, as well as their therapeutic implications.

## 1. Introduction

Since the end of the 19th century, plasma cells (PC) have been a major topic of research in order to understand their function and origin [1]. The hypothesis of PC lymphocytic genesis was first formulated in 1902 by Alexander Maximow in his unitarian theory of hematopoiesis [2]. Then, in 1947, Astrid Fagraeus demonstrated in vitro that PC are the antibody-secreting cells [3]. However, it was not until 1965 that Max Cooper and Robert Good demonstrated that PC derived from lymphocytes in the bursa of Fabricius (by definition B lymphocytes), found in all modern birds (Neorthis) [4].

It is now established that PC constitute the terminal stage of B lymphocyte differentiation and are the major players of the humoral immune response. Under pathological conditions, PC are at the center of allergic and autoimmune hypersensitivity reactions. Moreover, multiple myeloma (MM), a frequent hematologic cancer that in most cases remains untreated, is caused by malignant PC transformation and accumulation in the bone marrow [5]. In this context, many groups are investigating the modulation of the physiological differentiation of PC by epigenetic factors as well as their tumoral transformation.

Polycomb group (PcG) proteins are major epigenetic regulators of gene expression during development and cell fate choice. The first PcG component, the *Polycomb* gene, was discovered by Pamela Lewis in *Drosophila melanogaster* in 1947 [6]. A paradigm establishes that PcGs act as transcriptional repressors, although more recent observations have suggested that PcG might potentiate transcription. The two main PcG complexes are named polycomb repressive complex 1 (PRC1) and polycomb repressive complex 2 (PRC2), and function as multiprotein complexes that display strong evolutionary conservation [7].

In this review, we summarize the current knowledge on PcG protein implication in PC differentiation, myelomagenesis, and MM pathophysiology. Then, we discuss potential therapeutic options for patients with MM on the basis of these data.

## 2. PcG Complexes

**Figure 1:**
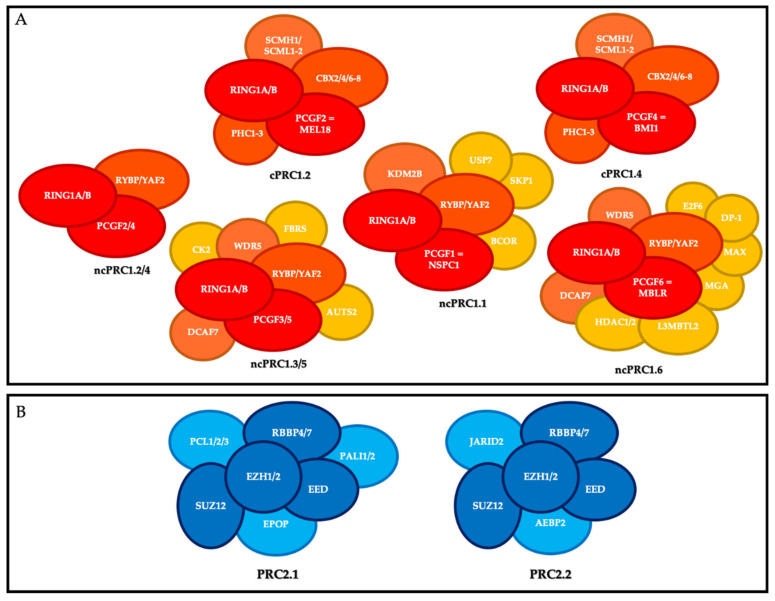
Polycomb repressive complexes (PRC). (**A**) Composition of canonical PRC1 (cPRC1) and non-canonical PRC1 (ncPRC1). Red, core members; orange, members that define the different canonical and non-canonical complexes; yellow, accessory factors. (**B**) Composition of PRC2. Dark blue, core members; light blue, members that define the different complexes.

PRC1 is composed of a core that includes the E3 ubiquitin ligase enzymes RING1A or RING1B, and one of the PCGF1-6 subunits. RING1 is the catalytic subunit that catalyzes the monoubiquitylation of lysine 119 of histone H2A (H2AK119ub1) on chromatin and interacts in a mutually exclusive manner with a chromobox protein (CBX 2, 4, 6–8) or RYBP (or its close homolog YAF2). On this basis, mammalian PRC1 complexes comporting a CBX subunit have been classified as canonical PRC1 (cPRC1), and PRC1 complexes containing RYBP or YAF2 have been classified as non-canonical PRC1 (ncPRC) [7]. Moreover, depending on the PCGF subunit associated with RING1A/B, eight different PRC1 complexes have been described and divided into canonical and non-canonical groups (also known as variants) [8] (Figure 1).

The canonical PRC1s (cPRC1s) are cPRC1.2 and cPRC1.4. In addition to RING1A or RING1B, their core contains MEL18 (PCGF2) and BMI-1 (PCGF4), respectively; one of the CBX2/4/6–8 proteins, which harbor the chromodomain allowing cPRC1 to recognize tri-methylation of lysine 27 of histone H3 (H3K27me3); and one of the three proteins PHC1-3 [9]. cPRC1 also includes the following accessory non-stoichiometric members: SCMH1, and SCMHL1/2 [10].

The non-canonical PRC1s (ncPRC1s) are ncPRC1.1, ncPRC1.2/4, ncPRC1.3/5, and ncPRC1.6. In addition to RING1 subunit, their cores include NSPC1 (PCGF1), PCGF2/4, PCGF3/5, and MBLR (PCGF6), respectively, and RYBP or YAF2. The ncPRC1 group includes many accessory members, particularly KDM2B and BCOR for ncPRC1.1; AUTS2 for PRC1.3/5; and HDAC1/2, E2F6, MAX and MGA for PRC1.6 [10].

PRC2 is composed of a core that includes the histone methyl transferases EZH1 or EZH2, which catalyze methylation of histone H3 at lysine 27 (H3K27me3) on chromatin via its SET domain, as well as its partners EED, SUZ12, and RBBP4/7, which are essential for its function. Depending on the members associated with this core, there are two main PRC2s: PRC2.1 (which includes EPOP, PALI1/2, and PCL1-3) and PRC2.2 (which includes AEBP2 and JARID2) [11].

**Figure 2:**
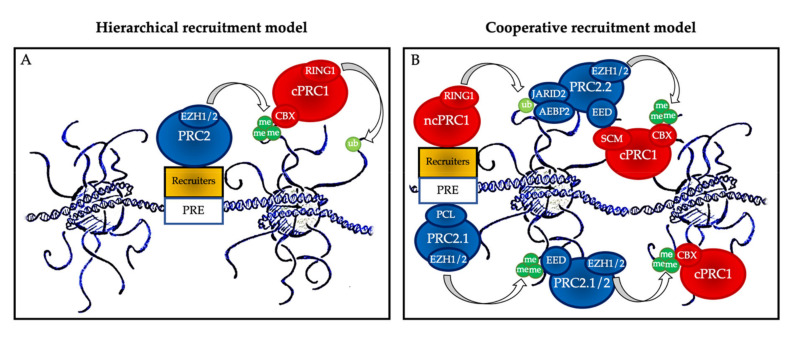
Polycomb group protein chromatin recruitment models. (**A**) Hierarchical recruitment model: PRC2 is recruited first and deposits H3K27me3 on chromatin via its catalytical subunit EZH1 or EZH2; then, canonical PRC1 (cPRC1) is recruited by a chromobox member CBX on the H3K27me3 mark and deposits H2AK119ub1 on chromatin via its catalytical subunit RING1. (**B**) Cooperative recruitment model: ncPRC1 complexes deposit H2AK119ub, which recruits PRC2.2 via its JARID2 and AEBP2 subunits. In parallel, PRC2.1 is recruited to unmethylated CpG island DNA via its PCL subunits. PRC2.1 and PRC2.2 complexes deposit H3K27me3, and this mark recruits both more copies of PRC2 and cPRC1. Mutual interactions between the core PRC2 member EED and the cPRC1 member SCM further stabilize their recruitment. PRE: polycomb responsive element (considered as CpG islands in mammals).

One of the important points in the biology of PcG proteins is that none of the core members of PRC1 or PRC2 can recognize specific DNA sequences on their own, and therefore they all need to be recruited by partners to regulate the specific expression of their target genes [8]. These partners include accessory proteins that bind unmethylated CG-rich sequences, histone marks, transcription factors, and RNAs, and much remains to be learnt about the precise mechanisms, cell type, and time-specificity of PcG recruitment at their targets [10,12,13] (Figure 2).

The historical hierarchical model described by Wang et al. in 2004 postulates that PRC2 is first recruited to chromatin and deposits H3K27me3. This epigenetic mark is then recognized by the chromodomain of CBX2/4/6–8, allowing recruitment of cPRC1 and the subsequent deposition of H2AK119ub1. According to this model, PRC1 is recruited in a PRC2-dependent manner. More recent data have allowed this model to be considerably refined. We now know that ncPRC1 binds independently on PRC2, either via proteins such as KDM2B (PRC1.1) [12], via interactions with ncRNAs such as Xist (PRC1.3 and PRC1.5) [14], or via transcription factors (TFs) [15]. ncPRC1 complexes deposit H2AK119ub, which recruits PRC2.2 via its JARID2 and AEBP2 subunits [16,17,18]. In parallel, PRC2.1 is recruited to unmethylated CpG island DNA via its PCL subunits [19]. Finally, PRC2 complexes deposit H3K27me3, and this mark recruits both more copies of PRC2 and cPRC1 [11]. Mutual interactions between these proteins further stabilize their recruitment. Therefore, a series of DNA-protein, RNA-protein, protein-protein, and protein-histone interactions lead to stable PcG protein recruitment to their target sites (Figure 2B). In this scenario, multiple signals, including PcG subunit, TF, or ncRNA abundance as well as CpG density and length can modulate PcG recruitment, offering multiple possibilities for regulation, leading to maintenance, stabilization, or displacement, depending on regulatory cues.

## 3. Lymphopoiesis and Plasma Cell Differentiation

The production of high affinity immunoglobulins (Igs; antibodies) is the critical point of the humoral immune response and the principle of vaccination strategies [20]. Antibody-secreting cells (ASC) include pre-plasmablasts (prePB), plasmablasts (PB), and PC (i.e., the final stages of B lymphocyte differentiation) [21].

B cell lymphopoiesis consists of several steps that take place in different anatomical compartments. Briefly, during the early medullary phase, hematopoietic stem cells (HSC) and lymphoid progenitors successively generate pro-B lymphocytes, pre-B lymphocytes, and immature B lymphocytes, independently of the antigen. During this phase, VDJ recombination of Ig heavy and light chains and selection of functional B lymphocytes take place [14,22]. Subsequently, immature B lymphocytes leave the medullary compartment to reach the spleen where, during a transitional phase, they become naive mature B lymphocytes that express IgM- and IgD-positive B cell receptors (BCRs) [23,24].

Naïve mature B lymphocytes have an immuno-surveillance role and begin to circulate in the follicles of the various secondary lymphoid organs (lymph nodes, spleen, and mucosa-associated lymphoid tissue). In these organs, the first contact between a naive B lymphocyte and its antigen triggers the primary humoral immune response. At this stage, a distinction is made between thymus-dependent (TD) antigens, which require the intervention of a follicular helper T lymphocyte (T_FH_, or more strictly a pre-T_FH_) to fully activate the mature B lymphocyte, and thymus-independent (TI) antigens, which can directly activate mature B cells by stimulating the toll-like receptor (TLR) pathway (TI type I antigens) or by cross-linking large numbers of BCRs simultaneously (TI type II antigens) [25].

The interaction between a mature B lymphocyte (through its BCR) and its specific antigen triggers two phenomena grouped under the term of “BCR triggering”: internalization of the BCR–antigen complex, and signal transduction through the BCR pathway. The BCR–antigen complex is internalized by a clathrin-dependent mechanism called receptor-mediated endocytosis and is driven to the endosomal pathway, where the antigen is loaded onto an MHC class II molecule that is then returned to the plasma membrane. In this process, the B lymphocyte assumes the role of professional antigen-presenting cell and becomes able to interact with a pre-T_FH_ lymphocyte. The BCR pathway includes the Lyn and Syk tyrosine kinases that induce the expression of co-stimulatory molecules and chemotactic receptors necessary for subsequent events [5,26].

At this stage, the B lymphocyte is not yet fully activated, but only pre-activated (primed), and can also be defined as B lymphoblast [27]. Upon contact with the antigen and activation of the BCR pathway, the naive mature B lymphocyte exits its quiescent state (reversible G0) and enters the G1 phase of the cell cycle, where it remains paused, awaiting an additional stimulus to complete its activation and proliferate [5]. This additional mitogenic stimulus can come from two different sources, depending on the nature of the antigen that triggered the pre-activation. TD antigens must interact with a pre-T_FH_ cell, whereas TI antigens have intrinsic activating activity [5]. Contact with pre-T_FH_ cells occurs in the B-T interface zone of secondary lymphoid organs through a structure known as the immune synapse, a specialized cell-cell junction organized in a bull’s eye pattern that was first described by Kupfer and his collaborators in 1998 [28] and reviewed by Dustin in 2015 [29].

**Figure 3:**
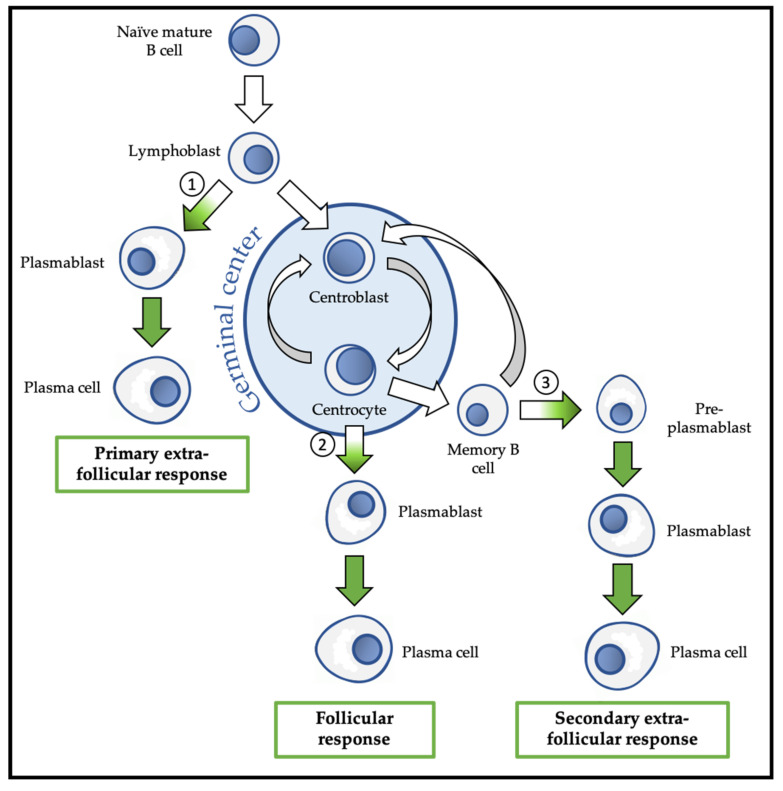
Overview of physiological differentiation of plasma cells (PC). Upon primary antigen contact, naïve mature B cells are activated and become lymphoblasts that can choose between two pathways: the primary extra-follicular response and the primary follicular response. In the primary extra-follicular response, lymphoblasts immediately differentiate into plasmablasts (PB) then into short-lived PC and produce immunoglobin M (IgM). In the primary follicular response, lymphoblasts enter a lymphoid follicle and form a germinal centre, while differentiating into centroblasts and then centrocytes. During the germinal centre reaction, immunoglobulin class switch recombination (CSR) and somatic hypermutation (SHM) take place. After selection, the centrocytes differentiate either into memory B cells, or into PB and then PC that produce IgG, IgA, or IgE. Upon secondary antigen contact, memory B cells are activated and can choose between a secondary extra-follicular response and a secondary follicular response. The secondary follicular response is similar to the primary follicular response and follows the same steps. In the secondary extra-follicular response, memory B cells immediately differentiate into pre-plasmablasts (pre-PB), PB, and then PC. The numbers indicate the cell fate decision points, where the antibody-secreting cell (ASC) program is switched on. Green arrows indicate cell differentiation steps where the ASC program takes place.

Following this activation, the B lymphoblast can choose between the follicular response and the extra-follicular response (Figure 3). The extra-follicular response is the only response to TI antigens and constitutes the early response to TD antigens. It allows the rapid production of short-lived ASC (PB and then PC) that secrete IgM with low affinity for the antigen [30].

The follicular response is specific to TD antigens and involves the formation of a secondary lymphoid follicle in which the processes of somatic hypermutation (SHM) and isotypic switching/class switch recombination (CSR) are carried out to generate antibodies with up to 30,000 times more affinity for the antigen and with a downstream isotype (IgG, IgA, or IgE) with new effector functions, respectively. The follicular response induces the differentiation of activated B lymphocytes into centroblasts and centrocytes, followed by the generation of PB that differentiate into short-lived (SLPC) and long-lived (LLPC) PC. LLPC can survive in specialized niches for decades [31]. The follicular response also generates memory B cells (MBC) [32,33].

MBC are the main players in the secondary humoral response that is triggered by a new contact with the same antigen. MBC activation can lead to their engagement in the extra-follicular response during which they differentiate into pre-PB, PB, and finally PC, or in a new follicular response with SHM, and possibly CSR [5].

Despite these various cell differentiation pathways, terminal B cell differentiation is transcriptionally regulated at two main developmental stages, each involving a specific network of transcriptional factors: the guardian network of the B phenotype, which includes PAX5, BACH2, and BCL6, and the vector network of the ASC phenotype, which includes PRDM1 (BLIMP1), IRF4, and XBP1 [20,30]. The complexity and beauty of PC differentiation lies in the regulation of the decision point between these two mutually exclusive networks that requires epigenetic mechanisms and PcG proteins.

The other critical point of the follicular response is the regulation of the genetic events that target the loci of the Ig genes during the germinal center reaction, namely, SHM and CSR, the understanding of which remains one of the main challenges in humoral immunology and pathological hematology. These two processes are orchestrated by the same enzyme, AID, a thymidine deaminase of the APOBEC family that exerts a physiological mutagenic activity exclusively in developing B lymphocytes [34,35]. PcG proteins have also been implicated in the regulation of AID-catalyzed reactions [36]. Current knowledge on this topic will be discussed in this review.

### 3.1. PcG Proteins and Early B Cell Differentiation

EZH2 is the catalytic subunit of PCR2. Besides its roles in myogenesis, adipogenesis, osteogenesis, neurogenesis, hepatogenesis, and epidermal differentiation, EZH2 also participates in hematopoiesis and lymphopoiesis [37,38]. Several studies on HSC have demonstrated that EZH2 stimulates HSC differentiation by inhibiting proliferation and apoptosis, without affecting their self-renewal capacity [39,40]. EZH2 is also required for VDJ recombination during early lymphopoiesis [41]. PCR1 role in hematopoiesis has been reviewed elsewhere [42,43].

### 3.2. PcG Proteins and the Primary Extra-Follicular Response

A subset of lymphoblasts activated by TI or TD antigens do not enter the follicle and immediately differentiate into PB [20]. Transcriptionally, a first decision point between the B cell and ASC networks occurs during the primary extra-follicular response (Figure 3). These PB immediately form para-follicular foci in the secondary lymphoid organs. After a proliferation phase, they rapidly differentiate into SLPC and produce IgM and IgD to quickly cope with the ongoing infection. They then undergo apoptosis after few days [30].

**Figure 4:**
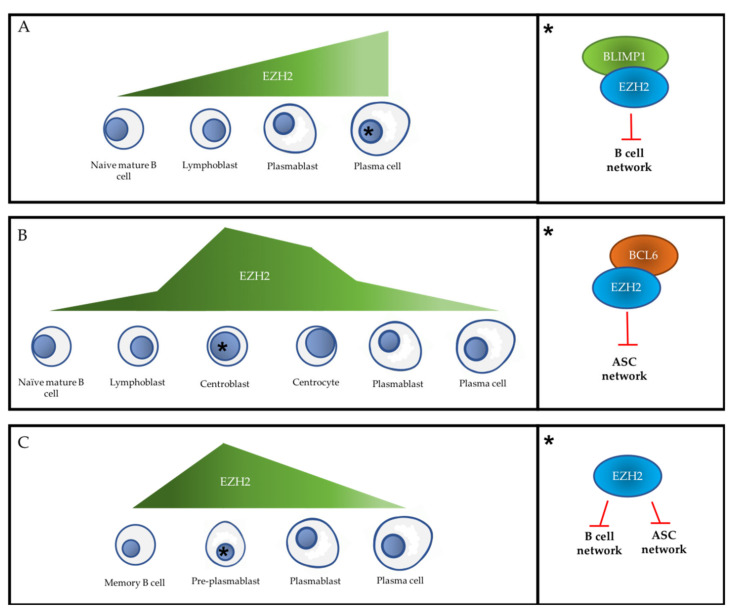
EZH2 expression level and its main cell fate control functions during PC differentiation. (**A**) Relative level of EZH2 expression during the primary extra-follicular response (left panel) and EZH2 main fate control function in primary extra-follicular PC (right panel). (**B**) EZH2 relative expression level during the follicular response (left panel) and EZH2 main fate control function in centroblasts (right panel). (**C**) Relative level of EZH2 expression during the secondary extra-follicular response (left panel) and EZH2 main fate control function in pre-PB (right panel). For each immunological response, the black asterisk indicates the cell step in which EZH2 expression level is maximal and refers to the right part of the figure. ASC: antibody-secreting cell. Red T-arrow represents repressive activity.

To date, EZH2 is the only PcG member that has been studied in the primary extra-follicular response, using a mouse model that cannot produce a follicular response due to a T_FH_ defect. In these mice, EZH2 is necessary in vivo for the generation of the extra-follicular response to TD and TI type I antigens [44]. Indeed, while naïve mature B cells do not express EZH2, its level of expression increases in lymphoblasts and reaches a maximum in extra-follicular ASCs [44] (Figure 4). Moreover, EZH2 depletion in these mice decreases B cell activation in lymph nodes and in spleen, as well as ASC proliferation and differentiation, and IgM production, without altering the mature B cell population [44].

**Figure 5:**
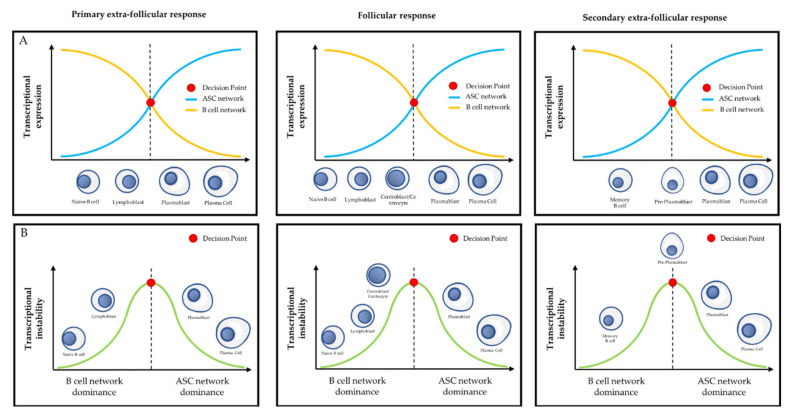
Transcriptional differentiation during PC differentiation. (**A**) Relationship between the antagonistic B cell transcriptional network and the ASC transcriptional network according to the cell differentiation step during the primary extra-follicular response (left), follicular response (middle), and secondary extra-follicular response (right). (**B**) Representation of the stability of the transcriptional network during PC differentiation. According to this model, naïve B cells and memory B cells represent the stable cell type that durably expresses B cell transcriptional network factors, and PC represents the stable cell type that durably expresses ASC transcriptional network factors. Other cell types (e.g., lymphoblasts, centroblasts, centrocytes, pre-PB, and PB) are transient and transcriptionally unstable, being are the site of active antagonism between the B cell and ASC transcriptional networks. The decision point represents the cell fate choice point where the transcriptional switch between the B cell network and the ASC network occurs, irreversibly inducing the activation of the ASC program and the extinction of the B cell program.

Mechanistically, in mice, EZH2 represses the B transcriptional network in ASC produced during the extra-follicular response by cooperating particularly with *Blimp-1* to target *Spib*, *Klf2*, *Tlr9*, and *Btg1* (Figure 4 and Figure 5). Many other B cell genes are significantly enriched in H3K27me3 or downregulated in the presence of EZH2 in these ASCs, such as *Cdkn1a*, *Nfkb1*, *Tnf*, *H2-Abl*, *Cd74*, *Bcl6*, *Klf4*, *Ifit3*, *Lta*, and *Slfn1* [44]. Of note, *Cdkn1a* encodes the p21^Cip^ [1] cell cycle inhibitor and is a known target of EZH2 during the germinal center (GC) reaction [45] and in other cell types [46]. Therefore, it has been suggested that in the absence of EZH2, ASC cannot proliferate due to their inability to suppress cell cycle inhibitors [44]. The phenotype and transcriptome of ASC produced at each mitosis were studied using an in vivo cell tracking model [47,48]. This analysis showed that in the absence of EZH2, the first three divisions after activation are normal, but most ASC become unable to continue to proliferate up to eight divisions. Moreover, the few ASC produced are metabolically dysfunctional, suggesting that EZH2 might be an upstream metabolic regulator in extra-follicular ASCs [44].

### 3.3. PcG Proteins and the Follicular Response

The interaction of pre-activated B lymphocytes with pre-T_FH_ lymphocytes in the B-T interface region of secondary lymphoid organs can direct B lymphocytes towards the follicular response that involves the generation of GC [49]. Chronologically, T_FH_ cells are the first to enter the primary lymphoid follicle. After about 24 h, fully activated B lymphoblasts join them in the middle of the follicle to generate an early GC. Then, the follicle takes the name of secondary lymphoid follicle. Within 72 h, the GC undergoes intense expansion and structural remodeling, leading to the formation of a light zone (LZ) and a dark zone (DZ), a phenomenon known as “GC polarization”. This expansion is generated by the intense proliferation of B lymphoblasts that gradually repel the naive mature B cells, which form the primary follicle, into the mantle area. Once the morphological reorganization is complete, the B lymphocytes in the DZ are called centroblasts and those in the LZ are called centrocytes. Together, these two cell populations are defined as GC B cells [50].

Centroblasts in the DZ undergo clonal expansion and SHM. The relatively quiescent centrocytes undergo a pseudo-Darwinian negative selection and are considered by some authors to be the stage when CSR takes place [50]. For the affinity maturation of immunoglobulins, GC B cells, particularly centroblasts, must maintain their respective phenotype long enough to undergo several rounds of SHM and division [47]. Furthermore, these cells have the unique ability to proliferate at high speed during SHM and CSR. Therefore, specific mechanisms to attenuate the DNA damage response, particularly cell cycle checkpoints, are required to tolerate the DNA lesions occurring during GC processes [51]. In this section, we describe the role played by PcG proteins, notably EZH2, in the control of GC events.

First, EZH2 is overexpressed by GC B cells compared to mature B cells and post-GC ASC generated by the primary humoral immune response [36,41,52,53]. Specifically, EZH2 expression level is highest in centroblasts, and dramatically decreases in centrocytes [52,53] (Figure 4 and Figure 5).

In mice, EZH2 is required for GC formation in vivo and promotes the proliferation of centroblasts and centrocytes ex vivo [51]. Its depletion leads to a 10-fold decrease in antibody production during the primary humoral immune response, lack of Ig-affinity maturation, and reduced MBC production [36,51]. All these findings indicate that EZH2 plays an important role in regulating the GC response, and is now considered a true master regulator of the GC phenotype.

In mice, depletion or inhibition of EZH2 methyltransferase activity reduces GC number and size in the spleen, and also clonal proliferation [36,51]. Conversely, EZH2 gain of function mutations induce an increase in GC number and size [51]. In addition, ex vivo studies on murine iGB cells (in vitro induced germinal center B cells) cells led to the discovery that EZH2 promotes the G1-S passage of GC B cells, independently of its repressive role towards cell cycle inhibitors (CDKN1A, CDKN1B, and CDKN2A). This is different from its role during the extra-follicular response [44].

Moreover, EZH2 has an anti-apoptotic effect in the GC that is not mediated by its role as p14^ARF^ inhibitor [54], and does not involve inhibition of the intrinsic apoptosis pathway [36].

At the transcriptional level, a second decision point between the B and ASC networks occurs during the transition between centroblasts/centrocytes and PB (Figure 3). The transcriptional regulation of GC B cells is clearly distinct from what is observed during the primary extra-follicular response. In murine iGC cells, EZH2 physically interacts with BCL6 and represses a subset of its target genes, including *Bcl2*, *Id2*, *Cdkn1b*, *Cdkn2a*, *Myb*, *Blimp-1*, and *Irf4* (Figure 4). This confers an ASC network repressor role on EZH2 that is different from its role as repressor of the B network described above [44]. Caganova and collaborators showed that EZH2 loss induces *Blimp-1* and *Irf4* transcription and ASC premature differentiation, a finding confirmed by Béguelin and collaborators in several human diffuse large B cell lymphoma (DLBC) cell lines (Pfeiffer, WSU-DLCL2, and Farage). In summary, EZH2 maintains the GC phenotype during the follicular response by suppressing the transcriptional program required to exit the GC and to initiate terminal differentiation, promoting B cell amplification before differentiation.

Finally, and surprisingly, Olivier Elemento’s team demonstrated ex vivo in human centroblasts and centrocytes that EZH2 is actively involved in the de novo establishment of bivalency at monovalent H3K4me3 promoters [51]. Bivalent promoters are characterized by the simultaneous presence of H3K27me3 and tri-methylation of lysine 4 of histone 3 (H3K4me3), two histone post-translational modifications that are usually mutually exclusive and antagonistic. During embryonic development, bivalent promoters are found at key cell fate genes that are paused while waiting for the cell to choose a differentiation path. After cell fate commitment, during specification and determination, these bivalent promoters are resolved towards stable expression (only H3K4me3) or sustained repression (only H3K27me3), which subsequently enables cell differentiation [55]. Therefore, the discovery of the de novo establishment of bivalent promoters by EZH2 is very interesting, especially because this phenomenon has not been described in the context of the extra-follicular (primary and secondary) responses. This feature gives centroblasts a unique pseudo-embryonic status during PC differentiation, perhaps facilitating the regulation of their fate choice between PB and MBC.

During the follicular response, SHM and CSR are mediated by targeting AID to the variable region of the Ig genes and switching sequences, respectively [40]. CSR is arguably the most studied process and has recently been reviewed elsewhere [56]. It is now clearly established that AID can catalyze the conversion of thymine to uracil only in a single-stranded DNA structure, and that this characteristic makes transcriptional activity essential at its target regions [56,57,58,59,60]. This CSR/SHM transcription coupling has led to many studies on R-loops and G-quadruplexes and to the emergence of several interesting hypotheses [56]. Nevertheless, AID targeting mechanism remains one of the most important questions to be elucidated in order to understand the factors leading to the CSR and SHM genesis and how its off-targets are physiologically avoided.

Interestingly, EZH2 loss induces a decrease in SHM without affecting CSR [36], thus raising questions about its crosstalk with AID. Because of its role, AID is a major determinant of genotoxic stress in the GC, and EZH2 protects centrocytes and centroblasts from AID-induced genotoxic stress [36]. Therefore, PcG proteins might play a role in the repair of AID-generated lesions. An interesting hypothesis is that EZH2, by globally repressing transcription in centrocytes and centroblasts, may contribute to AID targeting by limiting its off-target lesions [36].

Finally, and to conclude on the follicular response, it has been reported that the expression of EZH2 and EED in the GC is inversely correlated with that of BMI-1 and RING1B in centroblasts, centrocytes, and B cells of the mantle zone. EZH2 expression level in centrocytes is low, whereas BMI-1 and RING1B levels are high. Conversely, EZH2 expression level in mantle zone B cells and centroblasts is high, while BMI-1 and RING1B are weakly expressed [53,61]. These findings suggest a potential decoupling of PRC2 and PRC1 during the GC reaction, the effects of which on various cell processes (e.g., apoptosis, proliferation, differentiation) have not yet been explored. Further studies are needed on this issue, as well as on the role of the various PRC1s, which, to our knowledge, has never been investigated in the framework of the GC response.

### 3.4. PcG Proteins and the Secondary Extra-Follicular Response

When MBC meet their antigen for the second time, they can proliferate and differentiate much faster than mature naive B cells, despite a relatively comparable transcriptional program [5]. MBC can then progress towards the follicular response (described above) or the secondary extra-follicular response, in which they immediately differentiate into ASC, without going through the centroblast and centrocyte stages [62,63].

During ASC differentiation, a unique stage (i.e., pre-PB) has been described and characterized in vitro and in vivo [64,65,66], and the transcriptional decision point between the B and ASC networks might occur specifically at this stage (Figure 4 and Figure 5).

EZH2 (RNA and protein) is strongly overexpressed in pre-PB cells, remains high in PB, and disappears at the PC stage. Its expression level in these cell types is inversely correlated with that of EZH1, and this allows for the maintaining of a stable H3K27me3 level in the genome during PC differentiation while concomitantly finely regulating subgroups of key genes [67]. In pre-PB cells, EZH2 inhibits the B cell and ASC transcriptional networks, a new and unprecedented role compared with those described in the primary follicular and extra-follicular responses. This double EZH2 activity correlates with the fact that pre-PB cells co-express B and ASC factors at a low level. The inhibition of EZH2 catalytic activity during the extra-follicular secondary response accelerates PC differentiation by inducing the early overexpression of the ASC network and the premature inhibition of the B network and cell cycle genes [67].

Interestingly, in some genes that are overexpressed by pre-PB and PB cells relative to MBC and PC, EZH2 is located at their promoters in the absence of H3K27me3 [67]. Such promoters have been called EZH2_0_, and their discovery raises questions related to a possible role of EZH2 in activating their transcription. EZH2 might indirectly activate their transcription by repressing genes encoding microRNAs (miRNAs) that inhibit such genes. Direct activation by EZH2 would also be possible, although the molecular mechanisms at the basis of this function remain to be understood.

## 4. Multiple Myeloma

Multiple myeloma (MM) is a malignant hemopathy that affected nearly 230,000 people worldwide in 2015, with an incidence of around 130,000 new cases per year [68]. It is the second most frequent malignant hemopathy (10–13% in 2017) after non-Hodgkin lymphoma, and accounted for 1.7% of all cancers and 2% of all cancer deaths in 2017. MM is observed mainly in >50-year-old adults, and the average age at diagnosis is between 63 and 70 years.

MM development (i.e., myelomagenesis) is a multi-step process characterized by the appearance of genomic alterations and microenvironmental changes [69,70].

MM is a highly heterogeneous disease at the molecular and clinical levels [71,72,73,74]. Epigenetic modifications including DNA methylation, chromatin accessibility, and histone modifications have been reported in MM in association with pathogenic impact [75,76,77,78,79]. Moreover, the many epigenetic alterations observed in MM contribute to this biological heterogeneity and also to treatment resistance [80]. Due to their reversible nature, epigenetic alterations are particularly interesting for new targeted therapy strategies in MM [81,82]. PcG proteins are the subject of growing interest in MM with the hope of improving its poor prognosis.

### 4.1. PRC2 in Multiple Myeloma Pathophysiology

The PRC2 target genes, marked by H3K27me3 and previously identified in human embryonic fibroblasts, are under-expressed in MM [83]. Interestingly, these PRC2 target genes represent the bulk of genes that are downregulated in MGUS and MM, and the decrease in their expression strongly correlates with MM progression [83]. Therefore, PRC2 target genes are more strongly repressed in more advanced MM stages and in patients with poor prognosis [84].

Among all PcG proteins, EZH2 has been the most investigated in the context of MM pathophysiology. Its involvement in this malignancy has been suggested for over a decade. The arrival of pharmacological inhibitors, some specific and also usable in clinical practice, has intensified interest in EZH2 in the last 5 years. Studies on these inhibitors (UNC1999 [84,85,86], GSK343 [84,87,88], GSK126 [88,89,90], GSK2816126 [91], tazemetostat or EPZ-6438 or E-7438 [90,92,93,94], EPZ-005687 [95], OR-S1 and OR-S2 [96], and DNZep [83]) have already been discussed elsewhere [97].

Studies in mice xenografted with human MM cell lines (HMCLs) have shown that EZH2 is an oncogene in MM and that its pharmacological inhibition has an anti-tumor effect [85,92,98,99]. Its ectopic overexpression in tumor PC promotes their growth factor independence [99].

EZH2 expression is regulated at several levels. STAT3, c-MYC, and the NF-κB pathway can stimulate EZH2 transcription [100]. E2F1, which binds to the EZH2 promoter and induces its expression, is enriched in the nucleus of tumor PC due to the abnormally high basal activity of the PI3K/Akt pathway in MM. This explains why Akt inhibitors indirectly lower EZH2 level and have a synergistic effect with dual inhibitors that target both EZH2 and EZH1 [101]. Interestingly, this synergy is not observed with selective EZH2 inhibitors, suggesting a compensatory role for EZH1 in this context [101]. At the post-transcriptional level, loss of miR-26a, miR-101, let-7, and miR-138 increases EZH2 level in tumor PC [102,103,104]. Finally, EZH2 can be phosphorylated on serine 21 and consequently be inactivated by Akt. This promotes cell adhesion-mediated drug resistance in tumor PC in direct contact with marrow stromal cells [105].

Remarkably, EZH2 mutations have been described in other malignant hemopathies and in some HMCLs [73], but not in MM [84,95]. However, EZH2 is significantly overexpressed in tumor PC from the MGUS stage, and its expression, which correlates with the cell proliferation index, increases during the progression to SMM, reaching a maximum at the PCL stage [97,106,107]. EZH2 expression is particularly high in SP cells and in some tumor PC. Interestingly, the gene expression profile (GEP) of EZH2-overexpressing tumor PC resembles that of HMCLs [108]. High EZH2 expression at diagnosis is associated with poor prognosis [95], and EZH2 upregulation is usually accompanied by overexpression of EED and SUZ12. This suggests that in MM pathophysiology, EZH2 is active within the PRC2 [83].

EZH2 overexpression is necessary to induce the growth of HMCLs, especially those harboring mutations in N-RAS or K-RAS, suggesting context-specific functions [99]. EZH2 regulates the proliferation and survival of tumor PC by inducing the expression of genes that promote PB proliferation [93]. Its depletion or inhibition decreases the proliferation of some HMCLs and tumor PC in patients, induces their accumulation in the G0/G1 phase and apoptosis in a partially caspase-dependent mechanism [84,109], and decreases tumor mass in mouse models [83,92].

Intriguingly, EZH2 overexpression in MM is not associated with an overall increase in H3K27me3, but with its specific enrichment at some genes. In particular, in patients with the t (4;14) translocation and overexpression of the MMSET oncogene, the distribution of di-methylation of lysine 36 of histone 3 (H3K36me2) and H3K27me3 is strongly modified, with a significant H3K36me2 increase and a H3K27me3 decrease leading to the overexpression of some MM oncogenes [87,110,111]. EZH2 and H3K27me3 remain enriched at some loci that comprise c-MYC target genes and GC genes [87]. Interestingly, tumor PC harboring the t(4;14) translocation and overexpressing MMSET is more sensitive to EZH2 inhibition than isogenic cell lines that do not overexpress MMSET, suggesting a context-dependent oncogenic role for EZH2 [87].

After incubation with an EZH2 inhibitor, GEP analysis showed that the majority of deregulated genes are overexpressed; however, some genes are downregulated [93]. This suggests that EZH2 directly represses (via H3K27me3) several tumor suppressor genes and genes involved in differentiation, and indirectly activates oncogenes. EZH2 directly suppresses the pro-apoptotic genes *ID1*, *ID2*, *SOX2*, and *SORL1* [85] and some epithelial tumor suppressor genes such as *CDH1*, *EMP1*, *VCAN*, *EPHB2*, and *ENPP1*. This decreases MM cell adhesion capacity and alters their morphology, suggesting that EZH2 might be involved in MM cell dissemination mechanisms [92]. EZH2 also directly represses several genes involved in cell differentiation, senescence, and autophagy [97], as well as several cell cycle inhibitors, such as *CDKN2B* and *CDKN1A*. Interestingly, c-MYC also inhibits *CDKN2B* and *CDKN1A*, which creates a double mechanism of repressing these genes. Finally, EZH2 directly represses the tumor suppressor gene *RBPMS* that is involved in resistance to bortezomib, a proteasome inhibitor used for MM treatment [104].

Concomitantly, EZH2 indirectly activates the oncogenes CD69, JUNB, XBP1, IRF4, BLIMP1, and c-MYC [84,85,87]. Mechanistically, it is accepted that this indirect activation results from the direct repression of several miRNAs by EZH2. BLIMP1, IRF4, and XBP1 are miR-125a and miR-320c targets, while c-MYC is the target of miR-494. These tumor suppressor miRNAs are directly repressed by EZH2 in HMCLs and tumor PC [85,87,112]. EZH2 also directly represses miR-198, miR-601, miR125a-3p, and miR-320c, which are also inhibited by DNA methylation (i.e., the transfer of a methyl group to the carbon 5 position of cytosine to produce 5-methylcytosine, 5mC) in MM [112]. The potential cooperation of H3K27me3 and 5mC could thus provide a tight transcriptional repression, leading to a selection pressure that might favor cancer cell survival. Finally, EZH2 directly represses miR-138, which is an EZH2 inhibitor [104].

In conclusion, most GEP studies on genes deregulated in HMCLs after EZH2 inhibition indicate expression changes that promote cell cycle arrest, apoptosis, and repression of the c-MYC signature. However, the response to EZH2 inhibition is quite variable, independent of the effect on cell proliferation, partly reflecting the significant genetic and biological heterogeneity of the different cell lines tested, as well as the non-universal role of EZH2 in the various HMCLs [92].

It is important to note that, to date, sensitivity to EZH2 inhibition is not predicted by the extent of H3K27me3 reduction after treatment, by the initial EZH2 expression level, or by the mutational status of UTX, the main H3K27 demethylase, although UTX loss sensitizes MM cells to this inhibition [93]. Some HMCLs are resistant to EZH2 inhibitors, despite the overall H3K27me3 decrease. This could be explained by the presence of other genetic lesions that reduce the cellular dependence on EZH2, such as c-MYC translocations that can modify the mechanisms of c-MYC control. EZH2 might also act independently of its catalytic activity [97]. Our group identified a significant overlap between H3K27me3 level and DNA methylation at some genes in HMCLs that are resistant to EZH2 inhibition. We also found that sub-lethal doses of DNA methyltransferase inhibitors sensitize these resistant HMCLs to EZH2 inhibitors [93]. Conversely, EZH2 recruitment to its target genes has been reported to lead to DNA methylation [113]. Therefore, these two mechanisms of epigenetic repression might be linked.

Some other PRC2 members have been studied in the context of MM, but in much less detail. For example, the transcriptional regulation of EZH1 in MM is unclear [108], although like EZH2, EZH1 is overexpressed in SP cells. FOXO3 binds to the EZH1 promoter and stimulates its transcription. FOXO3 export from the nucleus by the PI3K/Akt pathway inhibits its transcriptional activity, explaining why Akt inhibition induces a compensatory EZH1 upregulation (at constant H3K27me3 levels) and EZH2 downregulation [101]. Therefore, as already demonstrated in acute myeloid leukemia [114], the concomitant inhibition of EZH1 and EZH2 appears to be an important element in MM and requires further study.

PHF19 (PCL3) is another PRC2 member that is overexpressed or duplicated in MM patients harboring trisomy of chromosome 9. Its expression correlates with MM progression [115]. Strong expression is found at relapse and is associated with a poor prognosis, with potential involvement in malignant progression and relapse [115,116]. In vitro, PHF19 is involved in HMCL proliferation and in their capacity to form colonies, and in vivo in myelomagenesis through its interaction with PRC2 [115]. In MM, PHF19 represses the CDKN1A/C cell cycle inhibitors as well as genes of the JAK-STAT interferon pathway [115]. Its depletion in several HMCLs (KMS11, MM1.S, U266, L363, NCI-H929, and RPMI1-8226) induces an increase in H3K36me2 and a global and non-specific genomic decrease in H3K27me3, with the exception of CpG-rich promoters [115]. This observation led to the hypothesis that PHF19, by stabilizing PRC2, is required for H3K27me3 spreading after the initial recruitment of PRC2 at CpG islands. In addition, it was noted that H3K27me3 distribution after PHF19 depletion is similar to that observed in other cancers in which lysine 27 of histone 3 is mutated into methionine (H3K27M, an oncogenic mutation). However, a recent study carried out by other authors on ARP-1 and OCI-My5 HMCLs reported that PHF19 promotes the phosphorylation-related inactivation of EZH2 by activating the PI3K/AKT pathway. This causes a decrease in H3K27me3 and H3K27me2 and then promotes expression of HIF-1α, Bcl-xL, and Mcl-1, thereby inducing MM cell proliferation and conferring drug resistance [116]. These contradictory data will need further investigations, but may imply that PHF19 could play a context-dependent regulation of PRC2 catalytical activity. To sum up, it seems that PRC2 deregulation, both positive and negative, may be involved in tumorigenesis. There is currently no pharmacological inhibitor of PHF19.

Finally, a recent genome-wide association study identified *JARID2* as a locus influencing susceptibility to MM [117].

On the basis of the growing body of data on PRC2 involvement in the MM pathophysiology, particularly its catalytic subunit EZH2, our group developed a theragnostic score called EZ-score, on the basis of the expression level of 15 genes that are sensitive to treatment with the EZH2 inhibitor EPZ-6438, associated with H3K27me3 and with a prognostic value in cohorts of patients with MM [93]. Patients with a high EZ-score have a poor prognosis and may benefit from treatment with an EZH2 inhibitor.

### 4.2. PRC1 in Multiple Myeloma Pathophysiology

BMI-1 is the most studied PRC1 member and the specificity and effects of several potential inhibitors, such as PTC209 [118,119], PTC596, and PTC028 [120], are currently being investigated in MM. RU-A1 is another potent BMI-1 inhibitor that has not yet been used in MM [121].

Initially, BMI-1 was described in mice as collaborating with c-MYC to induce lymphomagenesis [122]. In MM, BMI-1 is a potential oncogene that is overexpressed in primary tumor PC and in HMCLs [123,124,125]. Moreover, BMI-1 is strongly expressed in relapsed MM and this correlates with a shorter overall survival in patients with refractory MM treated with bortezomib or dexamethasone [118]. BMI-1 overexpression might be induced by c-MYC [126]. Interestingly, in breast cancer cell lines, BMI-1 activates the WNT pathway that in turns induces c-MYC activity, therefore maintaining a positive feedback loop between BMI-1 and c-MYC [127]. In MM, BMI-1 is also regulated by miR-203 [128], a miRNA suppressed by DNA hypermethylation in MM and chronic myeloid leukemia [129].

BMI-1 is necessary in vitro and in vivo for the clonogenic growth of myeloma cells and promotes their survival by repressing the pro-apoptotic *BIM* gene in some HMCL lines [130]. Contrary to what is observed in mouse embryonic fibroblasts [122], in MM, BMI-1 does not act by repressing the *CDNK2A* locus that encodes p16INK4 and p14ARF [130]. In other cancers, BMI-1 suppresses the tumor suppressor gene p19ARF, thus counteracting its overexpression induced by c-MYC [122]. It would be interesting to assess whether this also occurs in MM.

An interesting BMI-1 regulation axis through mucin 1 (MUC1) has recently been proposed by Tagde and collaborators [131]. MUC1 is a membrane heterodimer composed of a cytoplasmic portion (MUC1-C) and an ectodomain. It is always overexpressed in MM, and MUC1-C is an oncogene necessary for the proliferation and survival of myeloma cells [132,133,134]. In these cells, MUC1-C activates the WNT pathway [135,136], which promotes c-MYC transcription [137], and leads to the induction of BMI-1 expression and the creation of a MUC1–c-MYC–BMI-1 axis [131]. Other groups have also shown a physical interaction between BMI-1 and MUC1-C in other cancers, suggesting a putative post-transcriptional regulation of cPRC1 activity by MUC1-C [138]. Finally, MUC1-C also induces the expression of RING1A and RING1B in MM, but through different mechanisms—MYC-dependent for RING1B, and NF-kB p65-dependent for RING1A [131]. Interestingly, the WNT pathway is involved in the regulation of several PRC1 members. In addition, the WNT pathway is a direct target of PRC2 in MM and is important for the self-renewal maintenance of SP cells [108]. Although in many cancers the WNT/beta-catenin pathway is associated with tumor progression [139,140,141], it seems that in MM, its partial inhibition is necessary to ensure SP cell self-renewal, as indicated by the deleterious effect of beta-catenin overexpression on proliferation/viability of MM cells [108]. Understanding the dialogue between PRC1 and PRC2 to ensure this regulation might contribute to the revelation of the role of PcG proteins in MM pathophysiology.

### 4.3. PcG Proteins and Multiple Myeloma Tumor Microenvironment

Since the first description of MM in 1844, physicians have become aware of an unusual and unique type of bone disease that contributes significantly to MM morbidity through chronic osteodynia and spontaneous fractures [142]. Studies performed on the mechanistic basis of this bone disease have shown the accumulation of myeloma cells and osteoclasts at bone destruction sites. It is now established that myeloma cells locally produce osteoclastic activation molecules, such as IL-6 [143], TNFα [144], and MIP-α [145]. These cytokines are associated with cell adhesion [146,147], the deregulation of the RANK/RANKL/osteoprotegerin system [145,148,149], and an increase in the number and activity of osteoclasts. In addition, MM-associated bone disease is also characterized by inhibition of osteoblastic differentiation [150,151]. This leads to osteoblast/osteoclast decoupling and increased bone resorption with diffuse or multifocal osteolysis that can affect any bone in the body, but specifically the axial skeleton (pelvis, spine, skull) (for reviews on myeloma bone disease, see [142,152]).

**Figure 6:**
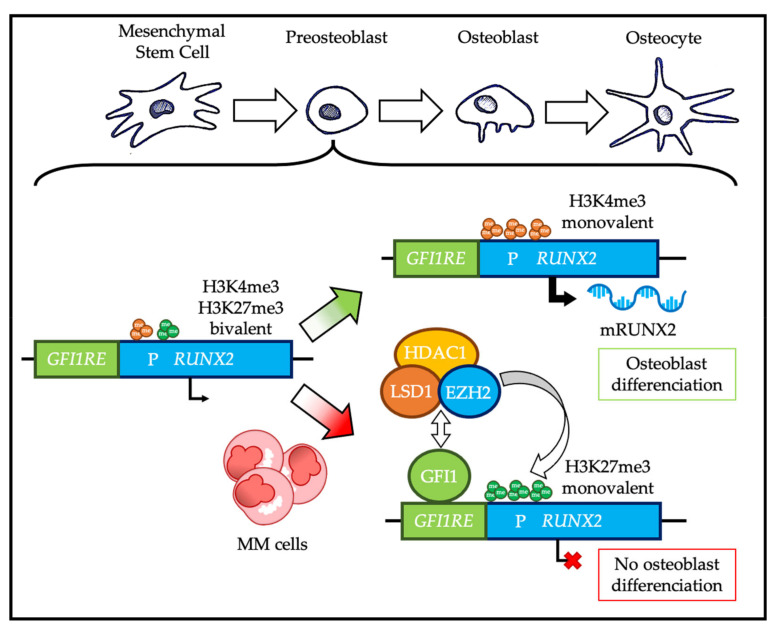
Role of EZH2 in multiple myeloma (MM) bone disease. Under physiological conditions, the *RUNX2* gene promoter is found in a bivalent state in osteoblastic progenitors; the normal resolution of this bivalency is oriented towards a H3K4me3 monovalent state, which allows the transcription of RUNX2 and then osteoblastic differentiation. However, in presence of MM cells, osteoblastic progenitors overexpress GFI1, which recruits EZH2, LSD1, and HDAC1 on RUNX2 promoter and induces a bivalent resolution oriented towards a H3K27me3 monovalent state and a transcriptional repression of RUNX2, resulting in an inhibition of osteoblastic differentiation. P: promoter; GFI1RE: GRI1 responsive element; mRUNX2: RUNX2 mRNA. Green arrow represents normal physiology and red arrow represents deregulations in MM malignancy.

It has been suggested that EZH2 overexpression in osteoblastic precursors may be involved in the development of bone lesions characteristic of MM. Myeloma cells induce expression of GFI1 in the osteoblastogenic bone marrow mesenchymal stem cells (BM-MSC) of the tumor microenvironment. In BM-MSC, GFI1 forms a complex with EZH2, HDAC1, and LSD1 and targets RUNX2, which is a central factor in BM-MSC differentiation into osteoblasts (Figure 6). EZH2 changes the status of the RUNX2 promoter from bivalent to repressed (only H3K27me3) [153]. Therefore, repression of RUNX2, as well as osteoprotegerin and osteocalcin by EZH2, directs BM-MSCs towards adipogenesis [154,155]. Importantly, it appears that this mechanism persists even after MM remission, and that inhibition of EZH2 or HDAC1 leads to RUNX2 re-expression and restores normal osteoblast differentiation [153].

In summary, EZH2 inhibition in the context of myeloma bone disease may inhibit bone degradation and promote bone reconstruction in patients with MM.

### 4.4. PcG Proteins and Drug Resistance in Multiple Myeloma

Several studies have demonstrated significant intra-clonal molecular heterogeneity, with the existence of different subclones already diagnosed with MM, and modifications in subclonal dominance during MM progression [156]. The subclones harbor different secondary cytogenetic lesions. These lesions are acquired randomly, due to genetic instability, and are then selected through a pseudo-Darwinian process that involves not only the tumor environment constraints but also the chemotherapy pressure [156]. They dictate the phenotype, chemoresistance, and clonogenic potential of each subclonal population. MM clonal evolution is not identical in all patients and at all stages of the disease, with different possible paths, ranging from the absence of genomic variations to a linear somatic evolution, or even somatic branching evolution [157].

MM treatment includes alkylating agents (melphalan, bendamustine, and cyclophosphamide), corticosteroids (dexamethasone and prednisone), immunomodulators or IMiD (thalidomide, lenalidomide, and pomalidomide), proteasome inhibitors (bortezomib, carfilzomib, and ixazomib), therapeutic monoclonal antibodies (elotuzumab, daratumumab, and isatuximab), an anti-tumor antibiotic (doxorubicin), a histone deacetylase inhibitor (panobinostat), or a spindle poison (vincristine) [158].

Despite promising clinical data, some patients do not respond to bortezomib at diagnosis, and only 25 to 30% still respond at MM relapse. This prompted several teams to study the biological bases of this clinical heterogeneity in which PcG proteins have been implicated on several occasions. Indeed, it has been shown that BMI-1 repression sensitizes myeloma cells to bortezomib [159]. In addition, strong EZH2 expression is predictive of a poor response to bortezomib, and ectopic expression of EZH2 in HMCLs confers resistance to this proteasome inhibitor [86]. Bortezomib has been reported to indirectly reduce EZH2 transcription and decrease E2F1 activity in myeloma cells [86]. In addition, the deregulation of the repression loop between EZH2 and miR-138 also participates in the resistance of myeloma cells to bortezomib by a mechanism that involves the tumor suppressor gene *RBPMS* [104]. However, these findings are still preliminary.

Epidrugs represent a significant therapeutic interest for overcoming drug resistance. For instance, pretreatment of HMCLs with an EZH2 inhibitor increases their sensitivity to panobinostat, an HDAC inhibitor, regardless of their initial sensitivity to such compound [90]. Moreover, the combined inhibition of EZH2 and BMI-1 has a synergistic effect in HMCLs and primary MM cells obtained from patients at MM diagnosis or relapse [119].

IMiDs such as lenalidomide promote binding of the Ikaros and Aiolos transcription factors to the E3 ubiquitin ligase cereblon (CRBN), leading to their ubiquitination and proteasomal degradation, associated with downregulation of IRF4 and MYC and reduced survival of MM cells [160,161]. Our team has demonstrated that the combination of EPZ-6438 with lenalidomide significantly decreases the levels of Ikaros, IRF4, and MYC proteins compared to lenalidomide or EPZ-6438 treatment alone [93]. Furthermore, EPZ-6438/lenalidomide combination strongly upregulates the B cell transcriptional network (PAX5, BACH2, BCL6), downregulates the ASC transcriptional network (PRDM1, IRF4), and results in a significant proliferation inhibition and quiescence induction in HMCL [93]. GEP data show that this combination induces significant and specific upregulation of a subset of genes targeted by PRC2, RB1, and DNA methylation, in association with significant downregulation of MYC target genes and cell proliferation program [93]. Interestingly, it was been recently shown that the combined inhibition of EZH2 and DNMTs re-sensitizes IMiD-resistant myeloma cells to lenalidomide and pomalidomide without altering the expression levels of the cereblon pathway members (CRBN, IKZF1, IKZF3, IRF4), by a mechanism that seems to involve SMAD3 [162]. Further work is needed to elucidate the basis of the lenalidomide–EZH2 inhibition synergy.

## 5. Concluding Remarks

We have described the multi-step gene expression regulation by PcG proteins during physiological PC differentiation and MM pathophysiology. Transcriptional regulation is at the top of this regulatory cascade. Indeed, transactivator and silencing factors play a relatively well-defined central role in PC differentiation, in particular via PRDM1, XBP1, IRF4, PAX5, BACH2, and BCL6 [30,163]. These factors, by definition capable of binding to chromatin, form antagonistic regulatory networks sensitive to environmental signals. However, they are only vectors, signal transducers of gene regulation. This means that to induce transcriptional expression or repression they must recruit cofactors (e.g., epigenetic regulators that chemically modify histones or DNA and/or chromatin remodelers that regulate nucleosome positioning and chromatin accessibility).

PcG proteins are major epigenetic regulators of cell fate during embryogenesis and differentiation of adult tissues. These proteins are also involved in cell transformation and have been studied in many cancers (for a review, see [164]). Their involvement in PC differentiation and MM pathophysiology is currently the subject of intense research, aimed at understanding the molecular mechanisms underlying these still poorly understood phenomena. Interestingly, during PC differentiation, EZH2 plays specific context-dependent roles depending on the humoral immune response phase. In PB and PC produced early by the primary extra-follicular response, it inhibits the B transcriptional network and promotes the ASC phenotype. Conversely, in GC centroblasts and centrocytes, EZH2 inhibits the ASC transcriptional network and maintains the B phenotype, allowing CSR and SHM. Finally, in pre-PB produced during the secondary extra-follicular response, EZH2 concomitantly inhibits the B and ASC transcriptional networks. These different EZH2 functions depending on the cell differentiation step add a layer of complexity to the study of PcG proteins during PC differentiation. Recent work carried out in *D. melanogaster* has brought new knowledge on the mechanism of regulation of gene expression by PcG proteins [165]. Specifically, during the course of *D. melanogaster* development and larval imaginal disc differentiation, PRC1 and PRC2 can be functionally uncoupled and PRC1 redeployed to new target genes that are enriched for cancer-related ontologies [165]. Furthermore, the role played by the numerous ncPRC1s found in mammals, distinct from that of cPRC1s, also opens new functional avenues to be explored.

On the pathological side, MM is a severe condition with poor prognosis and is still incurable in most cases. Many groups are interested in the role of PcG proteins in MM. However, further research is needed to better characterize the biological function of these epigenetic regulators of gene expression in a cancer context and also to identify new therapeutic avenues. Indeed, epigenetic mechanisms are characterized by their plasticity, which makes them targets of choice for developing new specific therapies. This also involves stratifying patients on the basis of biomarkers in order to identify MM patients that might benefit from epidrugs. Thus, targeting PcG proteins in MM constitutes a promising and innovative personalized therapeutic strategy. It is now necessary to define the most synergistic drug combination in order to improve the management of MM patients.

## References

[1] Ribatti D. (2017). The discovery of plasma cells: An historical note. Immunol. Lett..

[2] Maximow A.A. (1902). Chapter III-C. Experimentelle Untersuchungen über Entzündliche Neubildung von Bindegewebe.

[3] Fagraeus A. (1947). Plasma Cellular Reaction and its Relation to the Formation of Antibodies in vitro. Nature.

[4] Cooper M.D., Peterson R.D.A., Good R.A. (1965). Delineation of the thymic and bursal lymphoid systems in chicken. Nature.

[5] Cyster J.G., Allen C.D.C. (2019). B cell responses—Cell interaction dynamics and decisions. Cell.

[6] Lewis P.H. (1947). Melanogaster-New mutants: Report of Pamela H. Lewis. Dros. Inform. Serv..

[7] Chittock E.C., Latwiel S., Miller T.C.R., Müller C.W. (2017). Molecular architecture of polycomb repressive complexes. Biochem. Soc. Trans..

[8] Blackledge N.P., Rose N.R., Klose R.J. (2015). Targeting polycomb systems to regulate gene expression: Modifications to a complex story. Nat. Rev. Mol. Cell Biol..

[9] Ma R., Zhang Y., Sun T., Cheng B. (2014). Epigenetic regulation by polycomb group complexes: Focus on roles of CBX proteins. J. Zhejiang Univ. Sci. B.

[10] Schuettengruber B., Bourbon H.-M., Di Croce L., Cavalli G. (2017). Genome Regulation by Polycomb and Trithorax: 70 Years and Counting. Cell.

[11] Healy E., Mucha M., Glancy E., Fitzpatrick D.J., Conway E., Neikes H.K., Monger C., Van Mierlo G., Baltissen M.P., Koseki Y. (2019). PRC2.1 and PRC2.2 Synergize to Coordinate H3K27 Trimethylation. Mol. Cell.

[12] Blackledge N.P., Farcas A.M., Kondo T., King H.W., McGouran J.F., Hanssen L.L.P., Ito S., Cooper S., Kondo K., Koseki Y. (2014). Variant PRC1 Complex-Dependent H2A Ubiquitylation Drives PRC2 Recruitment and Polycomb Domain Formation. Cell.

[13] Simon J.A., Kingston R.E. (2013). Occupying chromatin: Polycomb mechanisms for getting to genomic targets, stopping transcriptional traffic, and staying put. Mol. Cell.

[14] Mårtensson I.-L., Almqvist N., Grimsholm O., Bernardi A.I. (2010). The pre-B cell receptor checkpoint. FEBS Lett..

[15] Yu M., Mazor T., Huang H., Huang H.-T., Kathrein K.L., Woo A.J., Chouinard C.R., Labadorf A., Akie T.E., Moran T.B. (2012). Direct Recruitment of Polycomb Repressive Complex 1 (PRC1) to Chromatin by Core Binding Transcription Factors. Mol. Cell.

[16] Kalb R., Latwiel S., Baymaz H.I., Jansen P.W.T.C., Müller C.W., Vermeulen M., Müller J. (2014). Histone H2A monoubiquitination promotes histone H3 methylation in Polycomb repression. Nat. Struct. Mol. Biol..

[17] Cooper S., Grijzenhout A., Underwood E., Ancelin K., Zhang T., Nesterova T.B., Anil-Kirmizitas B., Bassett A., Kooistra S.M., Agger K. (2016). Jarid2 binds mono-ubiquitylated H2A lysine 119 to mediate crosstalk between Polycomb complexes PRC1 and PRC2. Nat. Commun..

[18] Tamburri S., Lavarone E., Fernández-Pérez D., Conway E., Zanotti M., Manganaro D., Pasini D. (2020). Histone H2AK119 Mono-Ubiquitination Is Essential for Polycomb-Mediated Transcriptional Repression. Mol. Cell.

[19] Perino M., van Mierlo G., Loh C., Wardle S.M.T., Zijlmans D.W., Marks H., Veenstra G.J.C. (2020). Two Functional Axes of Feedback-Enforced PRC2 Recruitment in Mouse Embryonic Stem Cells. Stem Cell Rep..

[20] Shapiro-Shelef M., Calame K. (2005). Regulation of plasma-cell development. Nat. Rev. Immunol..

[21] Tellier J., Nutt S.L. (2019). Plasma cells: The programming of an antibody-secreting machine. Eur. J. Immunol..

[22] LeBien T.W., Tedder T.F. (2008). B lymphocytes: How they develop and function. Blood.

[23] Loder B.F., Mutschler B., Ray R.J., Paige C.J., Sideras P., Torres R., Lamers M.C., Carsetti R. (1999). B Cell Development in the Spleen Takes Place in Discrete Steps and Is Determined by the Quality of B Cell Receptor–Derived Signals. J. Exp. Med..

[24] Chung J.B., Silverman M., Monroe J.G. (2003). Transitional B cells: Step by step towards immune competence. Trends Immunol..

[25] Liu Y.-J., Zhang J., Lane P.J.L., Chan E.Y.-T., Maclennan I.C.M. (1991). Sites of specific B cell activation in primary and secondary responses to T cell-dependent and T cell-independent antigens. Eur. J. Immunol..

[26] Kurosaki T., Shinohara H., Baba Y. (2010). B Cell Signaling and Fate Decision. Annu. Rev. Immunol..

[27] Murphy K., Weaver C. (2016). Chapter 1-18: Lymphocytes activated by antigen proliferate in the peripheral lymphoid organs, generating effector cells and immunological memory. Janeway’s Immunobiology.

[28] Kupfer A., Singer S.J. (1989). The specific interaction of helper T cells and antigen-presenting B cells. IV. Membrane and cytoskeletal reorganizations in the bound T cell as a function of antigen dose. J. Exp. Med..

[29] Dustin M.L. (2014). The immunological synapse. Cancer Immunol. Res..

[30] Nutt S.L., Hodgkin P.D., Tarlinton D.M., Corcoran L.M. (2015). The generation of antibody-secreting plasma cells. Nat. Rev. Immunol..

[31] Nguyen D.C., Joyner C.J., Sanz I., Lee F.E.-H. (2019). Factors Affecting Early Antibody Secreting Cell Maturation Into Long-Lived Plasma Cells. Front. Immunol..

[32] Shlomchik M.J., Weisel F. (2012). Germinal center selection and the development of memory B and plasma cells. Immunol. Rev..

[33] Klein U., Dalla-Favera R. (2008). Germinal centres: Role in B-cell physiology and malignancy. Nat. Rev. Immunol.

[34] Muramatsu M., Sankaranand V.S., Anant S., Sugai M., Kinoshita K., Davidson N.O., Honjo T. (1999). Specific Expression of Activation-induced Cytidine Deaminase (AID), a Novel Member of the RNA-editing Deaminase Family in Germinal Center B Cells. J. Biol. Chem..

[35] Xu Z., Pone E.J., Al-Qahtani A., Park S.-R., Zan H., Casali P. (2007). Regulation of aicda expression and AID activity: Relevance to somatic hypermutation and class switch DNA recombination. Crit. Rev. Immunol..

[36] Caganova M., Carrisi C., Varano G., Mainoldi F., Zanardi F., Germain P.-L., George L., Alberghini F., Ferrarini L., Talukder A.K. (2013). Germinal center dysregulation by histone methyltransferase EZH2 promotes lymphomagenesis. J. Clin. Investig..

[37] Chou R.-H., Yu Y.-L., Hung M.-C. (2011). The roles of EZH2 in cell lineage commitment. Am. J. Transl. Res..

[38] Herviou L., Cavalli G., Cartron G., Klein B., Moreaux J. (2015). EZH2 in normal hematopoiesis and hematological malignancies. Oncotarget.

[39] Mochizuki-Kashio M., Mishima Y., Miyagi S., Negishi M., Saraya A., Konuma T., Shinga J., Koseki H., Iwama A. (2011). Dependency on the polycomb gene Ezh2 distinguishes fetal from adult hematopoietic stem cells. Blood.

[40] Xie H., Xu J., Hsu J.H., Nguyen M., Fujiwara Y., Peng C., Orkin S.H. (2014). Polycomb repressive complex 2 regulates hematopoietic stem cell maintenance and differentiation in a developmental stage-specific manner. Cell Stem Cell.

[41] Su I., Basavaraj A., Krutchinsky A.N., Hobert O., Ullrich A., Chait B.T., Tarakhovsky A. (2003). Ezh2 controls B cell development through histone H3 methylation and Igh rearrangement. Nat. Immunol..

[42] Vidal M., Starowicz K. (2017). Polycomb complexes PRC1 and their function in hematopoiesis. Exp. Hematol..

[43] Isshiki Y., Nakajima-Takagi Y., Oshima M., Aoyama K., Rizk M., Kurosawa S., Saraya A., Kondo T., Sakaida E., Nakaseko C. (2019). KDM2B in polycomb repressive complex 1.1 functions as a tumor suppressor in the initiation of T-cell leukemogenesis. Blood Adv..

[44] Guo M., Price M.J., Patterson D.G., Barwick B.G., Haines R.R., Kania A.K., Bradley J.E., Randall T.D., Boss J.M., Scharer C.D. (2018). EZH2 Represses the B Cell Transcriptional Program and Regulates Antibody-Secreting Cell Metabolism and Antibody Production. J. Immunol..

[45] Béguelin W., Rivas M.A., Calvo Fernández M.T., Teater M., Purwada A., Redmond D., Shen H., Challman M.F., Elemento O., Singh A. (2017). EZH2 enables germinal centre formation through epigenetic silencing of CDKN1A and an Rb-E2F1 feedback loop. Nat. Commun..

[46] Kim K.H., Roberts C.W.M. (2016). Targeting EZH2 in cancer. Nat. Med..

[47] Scharer C.D., Barwick B.G., Guo M., Bally A.P.R., Boss J.M. (2018). Plasma cell differentiation is controlled by multiple cell division-coupled epigenetic programs. Nat. Commun..

[48] Scharer C.D., Patterson D.G., Mi T., Price M.J., Hicks S.L., Boss J.M. (2020). Antibody-secreting cell destiny emerges during the initial stages of B-cell activation. Nat. Commun..

[49] Stebegg M., Kumar S.D., Silva-Cayetano A., Fonseca V.R., Linterman M.A., Graca L. (2018). Regulation of the Germinal Center Response. Front. Immunol..

[50] De Silva N.S., Klein U. (2015). Dynamics of B cells in germinal centres. Nat. Rev. Immunol..

[51] Béguelin W., Popovic R., Teater M., Jiang Y., Bunting K.L., Rosen M., Shen H., Yang S.N., Wang L., Ezponda T. (2013). EZH2 is required for germinal center formation and somatic EZH2 mutations promote lymphoid transformation. Cancer Cell.

[52] Velichutina I., Shaknovich R., Geng H., Johnson N.A., Gascoyne R.D., Melnick A.M., Elemento O. (2010). EZH2-mediated epigenetic silencing in germinal center B cells contributes to proliferation and lymphomagenesis. Blood.

[53] Van Galen J.C., Dukers D.F., Giroth C., Sewalt R.G.A.B., Otte A.P., Meijer C.J.L.M., Raaphorst F.M. (2004). Distinct expression patterns of polycomb oncoproteins and their binding partners during the germinal center reaction. Eur. J. Immunol..

[54] Bracken A.P., Kleine-Kohlbrecher D., Dietrich N., Pasini D., Gargiulo G., Beekman C., Theilgaard-Mönch K., Minucci S., Porse B.T., Marine J.-C. (2007). The Polycomb group proteins bind throughout the INK4A-ARF locus and are disassociated in senescent cells. Genes Dev..

[55] Mikkelsen T.S., Ku M., Jaffe D.B., Issac B., Lieberman E., Giannoukos G., Alvarez P., Brockman W., Kim T.-K., Koche R.P. (2007). Genome-wide maps of chromatin state in pluripotent and lineage-committed cells. Nature.

[56] Yu K., Lieber M.R. (2019). Current insights into the mechanism of mammalian immunoglobulin class switch recombination. Crit. Rev. Biochem. Mol. Biol..

[57] Bransteitter R., Pham P., Scharff M.D., Goodman M.F. (2003). Activation-induced cytidine deaminase deaminates deoxycytidine on single-stranded DNA but requires the action of RNase. Proc. Natl. Acad. Sci. USA.

[58] Chaudhuri J., Tian M., Khuong C., Chua K., Pinaud E., Alt F.W. (2003). Transcription-targeted DNA deamination by the AID antibody diversification enzyme. Nature.

[59] Sohail A., Klapacz J., Samaranayake M., Ullah A., Bhagwat A.S. (2003). Human activation-induced cytidine deaminase causes transcription-dependent, strand-biased C to U deaminations. Nucleic Acids Res..

[60] Ramiro A.R., Stavropoulos P., Jankovic M., Nussenzweig M.C. (2003). Transcription enhances AID-mediated cytidine deamination by exposing single-stranded DNA on the nontemplate strand. Nat. Immunol..

[61] Raaphorst F.M., van Kemenade F.J., Fieret E., Hamer K.M., Satijn D.P.E., Otte A.P., Meijer C.J.L.M. (2000). Cutting Edge: Polycomb Gene Expression Patterns Reflect Distinct B Cell Differentiation Stages in Human Germinal Centers. J. Immunol..

[62] Kurosaki T., Kometani K., Ise W. (2015). Memory B cells. Nat. Rev. Immunol..

[63] Weisel F., Shlomchik M. (2017). Memory B Cells of Mice and Humans. Annu. Rev. Immunol..

[64] Jourdan M., Caraux A., De Vos J., Fiol G., Larroque M., Cognot C., Bret C., Duperray C., Hose D., Klein B. (2009). An in vitro model of differentiation of memory B cells into plasmablasts and plasma cells including detailed phenotypic and molecular characterization. Blood.

[65] Jourdan M., Caraux A., Caron G., Robert N., Fiol G., Rème T., Bolloré K., Vendrell J.-P., Gallou S.L., Mourcin F. (2011). Characterization of a Transitional Preplasmablast Population in the Process of Human B Cell to Plasma Cell Differentiation. J. Immunol..

[66] Leung-Hagesteijn C., Erdmann N., Cheung G., Keats J.J., Stewart A.K., Reece D., Chung K.C., Tiedemann R.E. (2013). Xbp1s-Negative Tumor B Cells and Pre-Plasmablasts Mediate Therapeutic Proteasome Inhibitor Resistance in Multiple Myeloma. Cancer Cell.

[67] Herviou L., Jourdan M., Martinez A.-M., Cavalli G., Moreaux J. (2019). EZH2 is overexpressed in transitional preplasmablasts and is involved in human plasma cell differentiation. Leukemia.

[68] Cowan A.J., Allen C., Barac A., Basaleem H., Bensenor I., Curado M.P., Foreman K., Gupta R., Harvey J., Hosgood H.D. (2018). Global Burden of Multiple Myeloma. JAMA Oncol..

[69] Kuehl W.M., Bergsagel P.L. (2002). Multiple myeloma: Evolving genetic events and host interactions. Nat. Rev. Cancer.

[70] Barwick B.G., Gupta V.A., Vertino P.M., Boise L.H. (2019). Cell of Origin and Genetic Alterations in the Pathogenesis of Multiple Myeloma. Front. Immunol..

[71] Walker B.A., Leone P.E., Chiecchio L., Dickens N.J., Jenner M.W., Boyd K.D., Johnson D.C., Gonzalez D., Dagrada G.P., Protheroe R.K.M. (2010). A compendium of myeloma-associated chromosomal copy number abnormalities and their prognostic value. Blood.

[72] Zhan F., Huang Y., Colla S., Stewart J.P., Hanamura I., Gupta S., Epstein J., Yaccoby S., Sawyer J., Burington B. (2006). The molecular classification of multiple myeloma. Blood.

[73] Vikova V., Jourdan M., Robert N., Requirand G., Boireau S., Bruyer A., Vincent L., Cartron G., Klein B., Elemento O. (2019). Comprehensive characterization of the mutational landscape in multiple myeloma cell lines reveals potential drivers and pathways associated with tumor progression and drug resistance. Theranostics.

[74] Lohr J.G., Stojanov P., Carter S.L., Cruz-Gordillo P., Lawrence M.S., Auclair D., Sougnez C., Knoechel B., Gould J., Saksena G. (2014). Widespread genetic heterogeneity in multiple myeloma: Implications for targeted therapy. Cancer Cell.

[75] Walker B.A., Wardell C.P., Chiecchio L., Smith E.M., Boyd K.D., Neri A., Davies F.E., Ross F.M., Morgan G.J. (2011). Aberrant global methylation patterns affect the molecular pathogenesis and prognosis of multiple myeloma. Blood.

[76] Kaiser M.F., Johnson D.C., Wu P., Walker B.A., Brioli A., Mirabella F., Wardell C.P., Melchor L., Davies F.E., Morgan G.J. (2013). Global methylation analysis identifies prognostically important epigenetically inactivated tumor suppressor genes in multiple myeloma. Blood.

[77] Ordoñez R., Kulis M., Russiñol N., Chapaprieta V., Carrasco-Leon A., García-Torre B., Charalampopoulou S., Clot G., Beekman R., Meydan C. (2020). Chromatin activation as a unifying principle underlying pathogenic mechanisms in multiple myeloma. Genome Res..

[78] Agirre X., Castellano G., Pascual M., Heath S., Segura V., Bergmann A., Esteve A., Merkel A., Raineri E., Agueda L. (2015). Whole-epigenome analysis in multiple myeloma reveals DNA hypermethylation of B cell-specific enhancers. Genome Res..

[79] Jin Y., Chen K., De Paepe A., Hellqvist E., Krstic A.D., Metang L., Gustafsson C., Davis R.E., Levy Y.M., Surapaneni R. (2018). Active enhancer and chromatin accessibility landscapes chart the regulatory network of primary multiple myeloma. Blood.

[80] Alzrigat M., Párraga A.A., Jernberg-Wiklund H. (2018). Epigenetics in multiple myeloma: From mechanisms to therapy. Semin. Cancer Biol..

[81] De Smedt E., Lui H., Maes K., De Veirman K., Menu E., Vanderkerken K., De Bruyne E. (2018). The Epigenome in Multiple Myeloma: Impact on Tumor Cell Plasticity and Drug Response. Front. Oncol..

[82] Dupéré-Richer D., Licht J.D. (2017). Epigenetic Regulatory Mutations and Epigenetic Therapy for Multiple Myeloma. Curr. Opin. Hematol..

[83] Kalushkova A., Fryknäs M., Lemaire M., Fristedt C., Agarwal P., Eriksson M., Deleu S., Atadja P., Österborg A., Nilsson K. (2010). Polycomb Target Genes Are Silenced in Multiple Myeloma. PLoS ONE.

[84] Agarwal P., Alzrigat M., Párraga A.A., Enroth S., Singh U., Ungerstedt J., Österborg A., Brown P.J., Ma A., Jin J. (2016). Genome-wide profiling of histone H3 lysine 27 and lysine 4 trimethylation in multiple myeloma reveals the importance of Polycomb gene targeting and highlights EZH2 as a potential therapeutic target. Oncotarget.

[85] Alzrigat M., Párraga A.A., Agarwal P., Zureigat H., Österborg A., Nahi H., Ma A., Jin J., Nilsson K., Öberg F. (2016). EZH2 inhibition in multiple myeloma downregulates myeloma associated oncogenes and upregulates microRNAs with potential tumor suppressor functions. Oncotarget.

[86] Rizq O., Mimura N., Oshima M., Saraya A., Koide S., Kato Y., Aoyama K., Nakajima-Takagi Y., Wang C., Chiba T. (2017). Dual Inhibition of EZH2 and EZH1 Sensitizes PRC2-Dependent Tumors to Proteasome Inhibition. Clin. Cancer Res..

[87] Popovic R., Martinez-Garcia E., Giannopoulou E.G., Zhang Q., Zhang Q., Ezponda T., Shah M.Y., Zheng Y., Will C.M., Small E.C. (2014). Histone Methyltransferase MMSET/NSD2 Alters EZH2 Binding and Reprograms the Myeloma Epigenome through Global and Focal Changes in H3K36 and H3K27 Methylation. PLoS Genet..

[88] Ezponda T., Dupéré-Richer D., Will C.M., Small E.C., Varghese N., Patel T., Nabet B., Popovic R., Oyer J., Bulic M. (2017). UTX/KDM6A Loss Enhances the Malignant Phenotype of Multiple Myeloma and Sensitizes Cells to EZH2 inhibition. Cell Rep..

[89] Zeng D., Liu M., Pan J. (2016). Blocking EZH2 methylation transferase activity by GSK126 decreases stem cell-like myeloma cells. Oncotarget.

[90] Harding T., Swanson J., Van Ness B. (2018). EZH2 inhibitors sensitize myeloma cell lines to panobinostat resulting in unique combinatorial transcriptomic changes. Oncotarget.

[91] Yap T.A., Winter J.N., Giulino-Roth L., Longley J., Lopez J., Michot J.-M., Leonard J.P., Ribrag V., McCabe M.T., Creasy C.L. (2019). Phase I Study of the Novel Enhancer of Zeste Homolog 2 (EZH2) Inhibitor GSK2816126 in Patients with Advanced Hematologic and Solid Tumors. Clin. Cancer Res..

[92] Hernando H., Gelato K.A., Lesche R., Beckmann G., Koehr S., Otto S., Steigemann P., Stresemann C. (2016). EZH2 Inhibition Blocks Multiple Myeloma Cell Growth through Upregulation of Epithelial Tumor Suppressor Genes. Mol. Cancer.

[93] Herviou L., Kassambara A., Boireau S., Robert N., Requirand G., Müller-Tidow C., Vincent L., Seckinger A., Goldschmidt H., Cartron G. (2018). PRC2 targeting is a therapeutic strategy for EZ score defined high-risk multiple myeloma patients and overcome resistance to IMiDs. Clin. Epigenetics.

[94] Kurmasheva R.T., Sammons M., Favours E., Wu J., Kurmashev D., Cosmopoulos K., Keilhack H., Klaus C.R., Houghton P.J., Smith M.A. (2017). Initial Testing (Stage 1) of Tazemetostat (EPZ-6438), a Novel EZH2 Inhibitor, by the Pediatric Preclinical Testing Program. Pediatr. Blood Cancer.

[95] Pawlyn C., Bright M.D., Buros A.F., Stein C.K., Walters Z., Aronson L.I., Mirabella F., Jones J.R., Kaiser M.F., Walker B.A. (2017). Overexpression of EZH2 in multiple myeloma is associated with poor prognosis and dysregulation of cell cycle control. Blood Cancer J..

[96] Honma D., Kanno O., Watanabe J., Kinoshita J., Hirasawa M., Nosaka E., Shiroishi M., Takizawa T., Yasumatsu I., Horiuchi T. (2017). Novel orally bioavailable EZH1/2 dual inhibitors with greater antitumor efficacy than an EZH2 selective inhibitor. Cancer Sci..

[97] Alzrigat M., Jernberg-Wiklund H., Licht J.D. (2018). Targeting EZH2 in Multiple Myeloma—Multifaceted Anti-Tumor Activity. Epigenomes.

[98] Croonquist P.A., Linden M.A., Zhao F., Van Ness B.G. (2003). Gene profiling of a myeloma cell line reveals similarities and unique signatures among IL-6 response, N-ras-activating mutations, and coculture with bone marrow stromal cells. Blood.

[99] Croonquist P.A., Van Ness B. (2005). The polycomb group protein enhancer of zeste homolog 2 (EZH2) is an oncogene that influences myeloma cell growth and the mutant ras phenotype. Oncogene.

[100] Neo W.H., Lim J.F., Grumont R., Gerondakis S., Su I. (2014). c-Rel Regulates Ezh2 Expression in Activated Lymphocytes and Malignant Lymphoid Cells. J. Biol. Chem..

[101] Rizk M., Rizq O., Oshima M., Nakajima-Takagi Y., Koide S., Saraya A., Isshiki Y., Chiba T., Yamazaki S., Ma A. (2019). Akt inhibition synergizes with polycomb repressive complex 2 inhibition in the treatment of multiple myeloma. Cancer Sci..

[102] Pichiorri F., Suh S.-S., Ladetto M., Kuehl M., Palumbo T., Drandi D., Taccioli C., Zanesi N., Alder H., Hagan J.P. (2008). MicroRNAs regulate critical genes associated with multiple myeloma pathogenesis. Proc. Natl. Acad. Sci. USA.

[103] Seckinger A., MeiΔner T., Moreaux J., Benes V., Hillengass J., Castoldi M., Zimmermann J., Ho A.D., Jauch A., Goldschmidt H. (2015). miRNAs in multiple myeloma—A survival relevant complex regulator of gene expression. Oncotarget.

[104] Rastgoo N., Pourabdollah M., Abdi J., Reece D., Chang H. (2018). Dysregulation of EZH2/miR-138 axis contributes to drug resistance in multiple myeloma by downregulating RBPMS. Leukemia.

[105] Furukawa Y., Kikuchi J. (2016). Epigenetic mechanisms of cell adhesion-mediated drug resistance in multiple myeloma. Int. J. Hematol..

[106] Zhan F., Hardin J., Kordsmeier B., Bumm K., Zheng M., Tian E., Sanderson R., Yang Y., Wilson C., Zangari M. (2002). Global gene expression profiling of multiple myeloma, monoclonal gammopathy of undetermined significance, and normal bone marrow plasma cells. Blood.

[107] Zhan F., Tian E., Bumm K., Smith R., Barlogie B., Shaughnessy J. (2003). Gene expression profiling of human plasma cell differentiation and classification of multiple myeloma based on similarities to distinct stages of late-stage B-cell development. Blood.

[108] Nakagawa M., Fujita S., Katsumoto T., Yamagata K., Ogawara Y., Hattori A., Kagiyama Y., Honma D., Araki K., Inoue T. (2019). Dual inhibition of enhancer of zeste homolog 1/2 overactivates WNT signaling to deplete cancer stem cells in multiple myeloma. Cancer Sci..

[109] Zhao F., Chen Y., Li R., Liu Y., Wen L., Zhang C. (2010). Triptolide alters histone H3K9 and H3K27 methylation state and induces G0/G1 arrest and caspase-dependent apoptosis in multiple myeloma in vitro. Toxicology.

[110] Martinez-Garcia E., Popovic R., Min D.-J., Sweet S.M.M., Thomas P.M., Zamdborg L., Heffner A., Will C., Lamy L., Staudt L.M. (2011). The MMSET histone methyl transferase switches global histone methylation and alters gene expression in t(4;14) multiple myeloma cells. Blood.

[111] Kuo A.J., Cheung P., Chen K., Zee B.M., Kioi M., Lauring J., Xi Y., Park B.H., Shi X., Garcia B.A. (2011). NSD2 links dimethylation of histone H3 at lysine 36 to oncogenic programming. Mol. Cell.

[112] Alzrigat M., Jernberg-Wiklund H. (2017). The miR-125a and miR-320c are potential tumor suppressor microRNAs epigenetically silenced by the polycomb repressive complex 2 in multiple myeloma. RNA Dis..

[113] Viré E., Brenner C., Deplus R., Blanchon L., Fraga M., Didelot C., Morey L., Van Eynde A., Bernard D., Vanderwinden J.-M. (2006). The Polycomb group protein EZH2 directly controls DNA methylation. Nature.

[114] Fujita S., Honma D., Adachi N., Araki K., Takamatsu E., Katsumoto T., Yamagata K., Akashi K., Aoyama K., Iwama A. (2018). Dual inhibition of EZH1/2 breaks the quiescence of leukemia stem cells in acute myeloid leukemia. Leukemia.

[115] Ren Z., Ahn J.H., Liu H., Tsai Y.-H., Bhanu N.V., Koss B., Allison D.F., Ma A., Storey A.J., Wang P. (2019). PHF19 promotes multiple myeloma tumorigenicity through PRC2 activation and broad H3K27me3 domain formation. Blood.

[116] Yu T., Du C., Ma X., Sui W., Yu Z., Liu L., Zhao L., Li Z., Xu J., Wei X. (2020). Polycomb-like Protein 3 Induces Proliferation and Drug Resistance in Multiple Myeloma and Is Regulated by miRNA-15a. Mol Cancer Res..

[117] Mitchell J.S., Li N., Weinhold N., Försti A., Ali M., van Duin M., Thorleifsson G., Johnson D.C., Chen B., Halvarsson B.-M. (2016). Genome-wide association study identifies multiple susceptibility loci for multiple myeloma. Nat. Commun..

[118] Bolomsky A., Schlangen K., Schreiner W., Zojer N., Ludwig H. (2016). Targeting of BMI-1 with PTC-209 shows potent anti-myeloma activity and impairs the tumour microenvironment. J. Hematol. Oncol..

[119] Alzrigat M., Párraga A.A., Majumder M.M., Ma A., Jin J., Österborg A., Nahi H., Nilsson K., Heckman C.A., Öberg F. (2017). The polycomb group protein BMI-1 inhibitor PTC-209 is a potent anti-myeloma agent alone or in combination with epigenetic inhibitors targeting EZH2 and the BET bromodomains. Oncotarget.

[120] Bolomsky A., Muller J., Stangelberger K., Lejeune M., Duray E., Breid H., Vrancken L., Pfeiffer C., Hübl W., Willheim M. (2020). The anti-mitotic agents PTC-028 and PTC596 display potent activity in pre-clinical models of multiple myeloma but challenge the role of BMI-1 as an essential tumour gene. Br. J. Haematol..

[121] Bartucci M., Hussein M.S., Huselid E., Flaherty K., Patrizii M., Laddha S.V., Kui C., Bigos R.A., Gilleran J.A., El Ansary M.M.S. (2017). Synthesis and Characterization of Novel BMI1 Inhibitors Targeting Cellular Self-Renewal in Hepatocellular Carcinoma. Target. Oncol..

[122] Jacobs J.J.L., Scheijen B., Voncken J.-W., Kieboom K., Berns A., van Lohuizen M. (1999). Bmi-1 collaborates with c-Myc in tumorigenesis by inhibiting c-Myc-induced apoptosis via INK4a/ARF. Genes Dev..

[123] De Vos J., Thykjær T., Tarte K., Ensslen M., Raynaud P., Requirand G., Pellet F., Pantesco V., Rème T., Jourdan M. (2002). Comparison of gene expression profiling between malignant and normal plasma cells with oligonucleotide arrays. Oncogene.

[124] Chng W.J., Kumar S., VanWier S., Ahmann G., Price-Troska T., Henderson K., Chung T.-H., Kim S., Mulligan G., Bryant B. (2007). Molecular Dissection of Hyperdiploid Multiple Myeloma by Gene Expression Profiling. Cancer Res..

[125] Zhan F., Barlogie B., Arzoumanian V., Huang Y., Williams D.R., Hollmig K., Pineda-Roman M., Tricot G., van Rhee F., Zangari M. (2007). Gene-expression signature of benign monoclonal gammopathy evident in multiple myeloma is linked to good prognosis. Blood.

[126] Guo W.-J., Datta S., Band V., Dimri G.P. (2006). Mel-18, a Polycomb Group Protein, Regulates Cell Proliferation and Senescence via Transcriptional Repression of Bmi-1 and c-Myc Oncoproteins. MBoC.

[127] Cho J.-H., Dimri M., Dimri G.P. (2013). A Positive Feedback Loop Regulates the Expression of Polycomb Group Protein BMI1 via WNT Signaling Pathway. J. Biol. Chem..

[128] Wu S.-Q., Niu W.-Y., Li Y.-P., Huang H.-B., Zhan R. (2016). miR-203 inhibits cell growth and regulates G1/S transition by targeting Bmi-1 in myeloma cells. Mol. Med. Rep..

[129] Wong K.-Y., Liang R., So C.-C., Jin D.-Y., Costello J.F., Chim C.-S. (2011). Epigenetic silencing of MIR203 in multiple myeloma. Br. J. Haematol..

[130] Jagani Z., Wiederschain D., Loo A., He D., Mosher R., Fordjour P., Monahan J., Morrissey M., Yao Y.-M., Lengauer C. (2010). The Polycomb Group Protein Bmi-1 Is Essential for the Growth of Multiple Myeloma Cells. Cancer Res..

[131] Tagde A., Markert T., Rajabi H., Hiraki M., Alam M., Bouillez A., Avigan D., Anderson K., Kufe D. (2017). Targeting MUC1-C suppresses polycomb repressive complex 1 in multiple myeloma. Oncotarget.

[132] Treon S.P., Mollick J.A., Urashima M., Teoh G., Chauhan D., Ogata A., Raje N., Hilgers J.H., Nadler L., Belch A.R. (1999). Muc-1 core protein is expressed on multiple myeloma cells and is induced by dexamethasone. Blood.

[133] Kawano T., Ahmad R., Nogi H., Agata N., Anderson K., Kufe D. (2008). MUC1 oncoprotein promotes growth and survival of human multiple myeloma cells. Int. J. Oncol..

[134] (2007). MUC1 (EMA) expressing plasma cells in bone marrow infiltrated by plasma cell myeloma. Histol. Histopathol..

[135] Huang L., Ren J. (2003). MUC1 Cytoplasmic Domain Coactivates Wnt Target Gene Transcription and Confers Transformation. Cancer Biol. Ther..

[136] Rajabi H., Ahmad R., Jin C., Kosugi M., Alam M., Joshi M.D., Kufe D. (2012). MUC1-C Oncoprotein Induces TCF7L2 Transcription Factor Activation and Promotes Cyclin D1 Expression in Human Breast Cancer Cells. J. Biol. Chem..

[137] Tagde A., Rajabi H., Bouillez A., Alam M., Gali R., Bailey S., Tai Y.-T., Hideshima T., Anderson K., Avigan D. (2016). MUC1-C drives MYC in multiple myeloma. Blood.

[138] Hiraki M., Maeda T., Bouillez A., Alam M., Tagde A., Hinohara K., Suzuki Y., Markert T., Miyo M., Komura K. (2017). MUC1-C Activates BMI1 in human cancer cells. Oncogene.

[139] Polakis P. (2000). Wnt signaling and cancer. Genes Dev..

[140] Kikuchi A. (2003). Tumor formation by genetic mutations in the components of the Wnt signaling pathway. Cancer Sci..

[141] Le P., McDermott J.D., Jimeno A. (2015). Targeting the Wnt pathway in human cancers: Therapeutic targeting with a focus on OMP-54F28. Pharmacol. Ther..

[142] Terpos E., Ntanasis-Stathopoulos I., Gavriatopoulou M., Dimopoulos M.A. (2018). Pathogenesis of bone disease in multiple myeloma: From bench to bedside. Blood Cancer J..

[143] Tanaka Y., Abe M., Hiasa M., Oda A., Amou H., Nakano A., Takeuchi K., Kitazoe K., Kido S., Inoue D. (2007). Myeloma Cell-Osteoclast Interaction Enhances Angiogenesis Together with Bone Resorption: A Role for Vascular Endothelial Cell Growth Factor and Osteopontin. Clin. Cancer Res..

[144] Lam J., Takeshita S., Barker J.E., Kanagawa O., Ross F.P., Teitelbaum S.L. (2000). TNF-α induces osteoclastogenesis by direct stimulation of macrophages exposed to permissive levels of RANK ligand. J. Clin. Invest..

[145] Terpos E., Politou M., Viniou N., Rahemtulla A. (2005). Significance of macrophage inflammatory protein-1 alpha (MIP-1α) in multiple myeloma. Leuk. Lymphoma.

[146] Mori Y., Shimizu N., Dallas M., Niewolna M., Story B., Williams P.J., Mundy G.R., Yoneda T. (2004). Anti-α4 integrin antibody suppresses the development of multiple myeloma and associated osteoclastic osteolysis. Blood.

[147] Vanderkerken K., Medicherla S., Coulton L., Raeve H.D., Willems A., Lawson M., Camp B.V., Protter A.A., Higgins L.S., Menu E. (2007). Inhibition of p38α Mitogen-Activated Protein Kinase Prevents the Development of Osteolytic Bone Disease, Reduces Tumor Burden, and Increases Survival in Murine Models of Multiple Myeloma. Cancer Res..

[148] Giuliani N., Colla S., Sala R., Moroni M., Lazzaretti M., La Monica S., Bonomini S., Hojden M., Sammarelli G., Barillè S. (2002). Human myeloma cells stimulate the receptor activator of nuclear factor-κB ligand (RANKL) in T lymphocytes: A potential role in multiple myeloma bone disease. Blood.

[149] Delgado-Calle J., Anderson J., Cregor M.D., Hiasa M., Chirgwin J.M., Carlesso N., Yoneda T., Mohammad K.S., Plotkin L.I., Roodman G.D. (2016). Bidirectional Notch signaling and osteocyte-derived factors in the bone marrow microenvironment promote tumor cell proliferation and bone destruction in multiple myeloma. Cancer Res..

[150] Evans C.E., Ward C., Rathour L., Galasko C.B. (1992). Myeloma affects both the growth and function of human osteoblast-like cells. Clin. Exp. Metast..

[151] Terpos E., Berenson J., Raje N., Roodman G.D. (2014). Management of bone disease in multiple myeloma. Expert Rev. Hematol..

[152] Terpos E., Christoulas D., Gavriatopoulou M., Dimopoulos M.A. (2017). Mechanisms of bone destruction in multiple myeloma. Eur. J. Cancer Care.

[153] Adamik J., Jin S., Sun Q., Zhang P., Weiss K.R., Anderson J.L., Silbermann R., Roodman G.D., Galson D.L. (2017). EZH2 or HDAC1 Inhibition Reverses Multiple Myeloma-Induced Epigenetic Suppression of Osteoblast Differentiation. Mol. Cancer Res..

[154] Hemming S., Cakouros D., Vandyke K., Davis M.J., Zannettino A.C.W., Gronthos S. (2016). Identification of Novel EZH2 Targets Regulating Osteogenic Differentiation in Mesenchymal Stem Cells. Stem Cells Dev..

[155] Hemming S., Cakouros D., Codrington J., Vandyke K., Arthur A., Zannettino A., Gronthos S. (2017). EZH2 deletion in early mesenchyme compromises postnatal bone microarchitecture and structural integrity and accelerates remodeling. FASEB J..

[156] Fakhri B., Vij R. (2016). Clonal Evolution in Multiple Myeloma. Clin. Lymphoma Myeloma Leuk..

[157] Keats J.J., Chesi M., Egan J.B., Garbitt V.M., Palmer S.E., Braggio E., Van Wier S., Blackburn P.R., Baker A.S., Dispenzieri A. (2012). Clonal competition with alternating dominance in multiple myeloma. Blood.

[158] Kumar S.K., Rajkumar V., Kyle R.A., van Duin M., Sonneveld P., Mateos M.-V., Gay F., Anderson K.C. (2017). Multiple myeloma. Nat. Rev. Dis. Primers.

[159] Wu S.-Q., Xu Z.-Z., Niu W.-Y., Huang H.-B., Zhan R. (2014). shRNA-mediated Bmi-1 silencing sensitizes multiple myeloma cells to bortezomib. Int. J. Mol. Med..

[160] Bjorklund C.C., Lu L., Kang J., Hagner P.R., Havens C.G., Amatangelo M., Wang M., Ren Y., Couto S., Breider M. (2015). Rate of CRL4CRBN substrate Ikaros and Aiolos degradation underlies differential activity of lenalidomide and pomalidomide in multiple myeloma cells by regulation of c-Myc and IRF4. Blood Cancer J..

[161] Guirguis A.A., Ebert B.L. (2015). Lenalidomide: Deciphering mechanisms of action in myeloma, myelodysplastic syndrome and beyond. Curr. Opin. Cell Biol..

[162] Dimopoulos K., Søgaard Helbo A., Fibiger Munch-Petersen H., Sjö L., Christensen J., Sommer Kristensen L., Asmar F., Hermansen N.E.U., O’Connel C., Gimsing P. (2018). Dual inhibition of DNMTs and EZH2 can overcome both intrinsic and acquired resistance of myeloma cells to IMiDs in a cereblon-independent manner. Mol. Oncol..

[163] Shaffer A.L., Lin K.-I., Kuo T.C., Yu X., Hurt E.M., Rosenwald A., Giltnane J.M., Yang L., Zhao H., Calame K. (2002). Blimp-1 Orchestrates Plasma Cell Differentiation by Extinguishing the Mature B Cell Gene Expression Program. Immunity.

[164] Chan H.L., Morey L. (2019). Emerging Roles for Polycomb-Group Proteins in Stem Cells and Cancer. Trends Biochem. Sci..

[165] Loubière V., Delest A., Thomas A., Bonev B., Schuettengruber B., Sati S., Martinez A.-M., Cavalli G. (2016). Coordinate redeployment of PRC1 proteins suppresses tumor formation during Drosophila development. Nat. Genet..

